# The Bonebridge BCI 602 Active Transcutaneous Bone Conduction Implant in Children: Objective and Subjective Benefits

**DOI:** 10.3390/jcm10245916

**Published:** 2021-12-16

**Authors:** Katarzyna B. Cywka, Henryk Skarżyński, Bartłomiej Król, Piotr H. Skarżyński

**Affiliations:** 1 Otorhinolaryngosurgery Clinic, World Hearing Center, Institute of Physiology and Pathology of Hearing, 02-042 Warsaw, Poland; k.cywka@ifps.org.pl (K.B.C.); b.krol@ifps.org.pl (B.K.); 2 Institute of Sensory Organs, 05-830 Warsaw, Poland; skarzynski.henryk@ifps.org.pl; 3 World Hearing Center, Department of Teleaudiology and Screening, Institute of Physiology and Pathology of Hearing, 02-042 Warsaw, Poland; 4 Heart Failure and Cardiac Rehabilitation Department, Second Faculty of the Medical University of Warsaw, 02-091 Warsaw, Poland

**Keywords:** Bonebridge, hearing implant, hearing loss, bone conduction, children, congenital ear deformities, hearing rehabilitation

## Abstract

Background: the Bonebridge hearing implant is an active transcutaneous bone conduction implant suitable for various types of hearing loss. It was first launched in 2012 as the BCI 601, with a newer internal part (BCI 602) released in 2019. With the new size and shape, the BCI 602 can be used in patients previously excluded due to insufficient anatomical conditions, especially in patients with congenital defects of the outer and middle ear. Objectives: the purpose of this study is to evaluate the objective and subjective benefits of the new Bonebridge BCI 602 in children who have hearing impairment due to conductive or mixed hearing loss. Safety and effectiveness of the device was assessed. Methods: the study group included 22 children aged 8–18 years (mean age 14.7 years) who had either conductive or mixed hearing loss. All patients were implanted unilaterally with the new Bonebridge BCI 602 implant. Pure tone audiometry, speech recognition tests (in quiet and noise), and free-field audiometry were performed before and after implantation. Word recognition scores were evaluated using the Demenko and Pruszewicz Polish Monosyllabic Word Test, and speech reception thresholds in noise were assessed using the Polish Sentence Matrix Test. The subjective assessment of benefits was carried outusing the APHAB (Abbreviated Profile of Hearing Aid Benefit) questionnaire. Results: after implantation of the Bonebridge BCI 602 all patients showed a statistically significant improvement in hearing and speech understanding. The mean word recognition score (WRS) changed from 12.1% before implantation to 87.3% after 6 months. Mean speech reception threshold (SRT) before implantation was +4.79 dB SNR and improved to −1.29 dB SNR after 6 months. All patients showed stable postoperative results. The APHAB questionnaire showed that difficulties in hearing decreased after implantation, with a statistically significant improvement in global score. Pre-operative scores (M = 35.7) were significantly worse than post-operative scores at 6 months (M = 25.7). Conclusions: the present study confirms that the Bonebridge BCI 602 is an innovative and effective solution, especially for patients with conductive and mixed hearing loss due to anatomical ear defects. The Bonebridge BCI 602 system provides valuable and stable audiological and surgical benefits. Subjective assessment also confirms the effectiveness of the BCI 602. The BCI 602 offers the same amplification as the BCI601, but with a smaller size. The smaller dimensions make it an effective treatment option for a wider group of patients, especially children with congenital defects of the outer and middle ear.

## 1. Introduction

Children with conductive or mixed hearing loss have limited options available for hearing rehabilitation, yet in the pediatric population, early intervention is crucial for auditory and language development [1]. Many patients who suffer from these types of hearing loss may not be able to use conventional hearing aids because of medical or anatomical conditions or may be dissatisfied with them. Often, middle ear surgery is not indicated in this group of patients [2].

An increasing number of patients have congenital defects of the outer and middle ear. Congenital malformation of the external and middle ear is not uncommon in clinical settings, and the average incidence of ear malformations is estimated to be 1:10,000 to 1:20,000. Unilateral defects occur more often than bilateral, with approximately 10% of patients suffering from bilateral malformations [3]. Bone conduction hearing aids (BCHAs) and bone conduction implants (BCIs) are alternatives for treating these groups of patients [4]. Bone conduction hearing aids (BCHAs) have recognized limitations: constant pressure on the skull, poor sound quality, inconvenience, unwanted visibility for older children and adolescents, and limited gain (in comparison to implantable devices). A better solution, as demonstrated by many studies, is the use of a BCI. Bone conduction hearing systems have become the standard solution for patients suffering from conductive, mixed, and unilateral hearing loss [5]. Currently, there are many bone conduction devices available on the market. Knowledge of the capabilities and limitations of these devices is essential in order to select the best solution for the patient, especially for a child [6,7,8,9].

### 1.1. Bone Conduction Hearing Systems

Bone conduction (BC) has been known for a long time. The phenomenon was first described as early as the 16th century and since then, due to numerous scientific studies, various devices that improve hearing using bone conduction have been developed. Bone conduction occurs when the bone of the skull transmits vibration directly to the cochlea, with the intensity of the vibration depending on the frequency of the incoming sound and the structure of the skull itself.

Percutaneous bone conduction implants (BCIs) provide significant audiological gain but are associated with a high rate of complications [10,11,12,13]. It has also been reported that the performance of active transcutaneous bone conduction hearing implant is superior to passive skin-driven BC devices with implanted magnets (which suffer a transcutaneous signal attenuation of 10–15 dB) because the signal in an active transcutaneous BC implant is independent of the thickness of the skin and hair.

Hougaard et al [14] reported that the functional gain in conductive or mixed hearing loss patients when using BAHA Attract was 19.8 dB, which is much lower than the improvement reported when using the BB BCI 602 implant. The disadvantages of percutaneous systems are skin reactions to the external abutment (with high infection rates, especially in children); hypertrophic scarring with skin overgrowth; and fixture losses, possibly leading to revision surgery or even explantation. Transcutaneous technology avoids the typical complications involved in percutaneous systems. The complications reported in the literature have been skin reactions from Holgers Grade 2 to 4 (ranging from 2.4 to 38.1%) and failure of osseointegration (ranging up to 18% in adult and mixed populations, and 14.3% in pediatric populations). The rate of revision surgery ranges up to 34.5% in adult and mixed populations and up to 44.4% in pediatric patients, whereas the total rate of implant loss ranges up to 17.4% in adult and mixed populations and 25% in pediatric patients.

Details of BCI602 bone-anchored hearing aid implantation procedures were retrieved from the departmental database. The overall complication rate was 23.9%, and the rate of revision surgery was 12.1%. Some publications show a high percentage of postoperative complications after the use of percutaneous implants, with one report that “A total of 58.8% of patients with Connect surgeries had complications within a year and 82.4% had a complication by their last follow-up” [10,11,12,13,15,16,17]. Active transcutaneous BCIs have good functional outcomes, directly vibrating the temporal bone for optimal sound quality and more powerful amplification while avoiding the complications associated with percutaneous abutment (skin reaction, skin growth over the abutment, and wound infection). The choice of a particular device depends on the patient’s audiological results, anatomy, and health. Each case needs to be considered individually [6,7,8,9].

### 1.2. BonebridgeDescription

The BonebridgeBCI601(Med-El, Innsbruck, Austria) was first implanted in 2011 as part of a clinical trial and was launched onto the EU market in September 2012 [18]. The Bonebridge consists of an external part (audio processor) and an internal part (bone-conduction floating mass transducer (BC-FMT) that is surgically implanted into the skull in either the transmastoid, retrosigmoid, or middle fossa regions. The external component digitally processes the sound and sends information through the coil to the implantable internal part, which applies vibrations directly to the bone and cochlea. The external component is small and contains a processor with microphone, magnet, and battery. The Bonebridge is intended for adults and for children over 5 years old.

**Figure 1:**
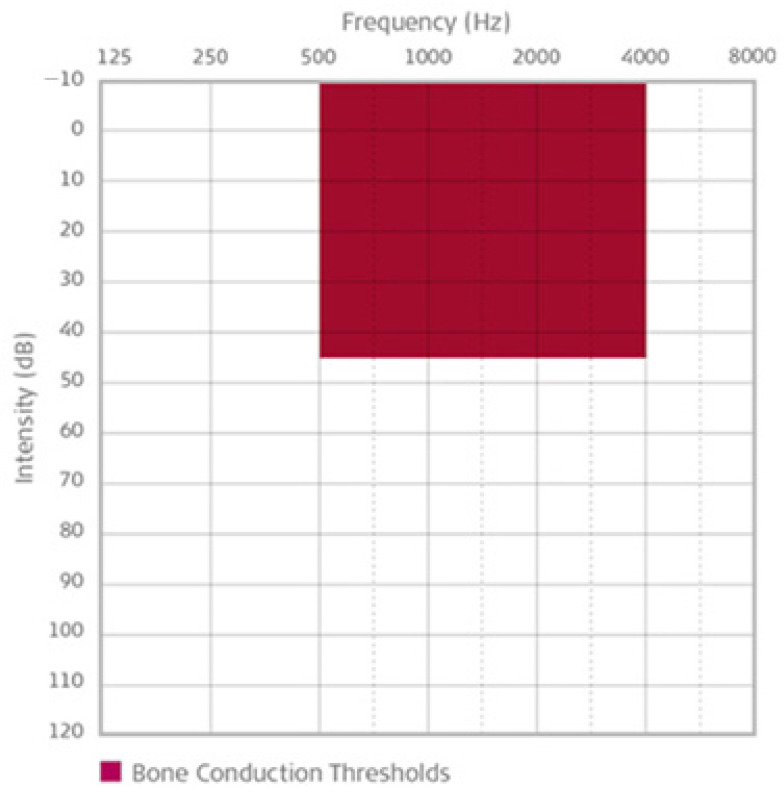
Indication range for bone conduction thresholds for the Bonebridge implant [19].

Indications recommended by Med-El are: conductive or mixed mild-to-moderate hearing loss; pure tone average (PTA) BC threshold (measured at 0.5, 1, 2, 3, and 4 kHz) ≥ 45 dB HL (Figure 1);profound sensorineural hearing loss in one ear and normal hearing in the opposite ear; and air conduction hearing thresholds in the hearing ear or sensorineural hearing loss in one ear and normal hearing in the opposite ear; and air conduction dual medical history has to be assessed to check for the following: active infection; cholesteatoma; revision tympanoplasty; ear canal stenosis or chronically draining ears where conventional hearing aids are not suitable; and otosclerosis or tympanosclerosis that cannot be rectified to a sufficient extent by surgery.

When there is an issue with treatment after cholesteatoma, it is still possible to apply a BCI, but before hand it is necessary to obliterate the cavity with bioactive glass [19,20]. On the other hand, favorable indications include wearing conventional hearing aids; congenital malformations in which the ear canals are absent; sudden deafness or other reasons that cause severe to profound sensorineural hearing loss on one side; anatomy that allows appropriate placement of the Bonebridge implant as determined by a CT scan; absence of retrocochlear and central auditory disorders; and psychological and emotional stability with realistic expectations of the benefits and limitations of an implant [21].

### 1.3. The New BonebridgeBCI 602

**Figure 2:**
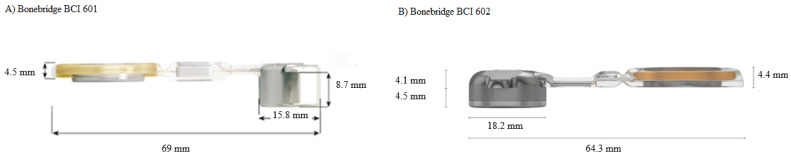
Bonebridge implants; (**A**)—First-generation (BCI 601); (**B**)—Second-generation (BCI 602) [21].

In 2019, Med-El introduced the next generation of their active bone conduction implant, the Bonebridge BCI 602.The main difference between the BCI601 (first generation) and the BCI602 (second generation) is the different transducer design (Figure 2).

**Figure 3:**
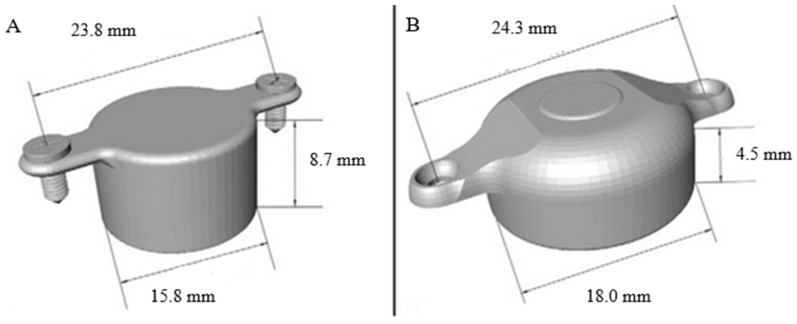
(**A**)—Dimensions of the BCI 601; (**B**)—dimensions of the BCI 602 [14].

The most significant difference between the two models is the shape and size of the internal part, where the thickness of the BC-FMT has narrowed from 8.7 to 4.5 mm (Figure 3).

This is a considerable advantage and gives opportunities for using it on patients where it was impossible to use the first-generation implant due to limited anatomical conditions (the internal part was too large). The upgrade gives the same power output for effective amplification, but with nearly 50% less drilling depth. The Bonebridge BCI 602 has the same audiological and medical criteria as the first-generation device [22,23].

Surgeons have mentioned several advantages of the new BCI 602, but the major one is flexible surgical placement. There is a flexible transition between the receiver coil and the floating mass transducer which allows it to bend up to 90° in either lateral direction. The transition can also bend medially up to 30° to accommodate curvature of the skull. A smaller drilling depth reduces drilling time and reduces the likelihood of dura exposure, decreasing surgical time significantly; in addition, self-drilling screws simplify handling [22,24,25].

This study assessed the effectiveness of the new Bonebridge BCI 602 system. The aim was to evaluate the surgical, audiological, and subjective benefits in 22 pediatric patients with conductive or mixed hearing loss after implantation.

## 2. Materials and Methods

### 2.1. Study Design and Participants

From February 2020 to March 2021, 22 patients with conductive or mixed hearing loss due to various medical reasons underwent unilateral BCI602 implantation. The patients were selected according to the following criteria: older than 5 years of age, ear reconstruction had been carried out and was complete; good cooperation in audiological tests; hearing thresholds accorded with the manufacturer’s suggested criteria; bone-conduction thresholds over the last 12 months were stable; appropriate anatomical conditions confirmed by a CT scan; and patients and their parents had realistic expectations of the benefits and limitations of the Bone bridge. The side of operation was selected based on the following considerations: if there was a bilateral disorder, the worse ear was selected based on comparison of hearing loss in the two ears. Alternatively, if there were no significant differences in temporal bone structure, if hearing loss in both ears was the same, and if a preoperative hearing test indicated the same benefits from a bone conduction hearing aid, then the patient could choose the side. In the diagnostic process, all patients had bone devices simulated using a bone hearing aid on a soft-band to estimate the real and possible benefits of implantation. Before the operation, all patients underwent a CT scan of the temporal bone and a detailed assessment of anatomical conditions was carried out with Otoplan software (Med-El, Innsbruck, Austria) [26,27]. Some 4 weeks (±1 week) after the operation, the device was activated and the settings adjusted according to the audiometric test results, vibrogram results, and the patient’s subjective assessment and comfort. All patients were provided with Samba (Med-El) processors, and the Symfit 7.0 program (Med- El, Innsbruck, Austria) was used for fitting. All fittings were performed by two experienced audiologists. The strength of the sound processor’s magnet was adjusted to provide a firm hold while avoiding skin compression.

The study protocol was approved by the Institutional Review Board of the Institute of Physiology and Pathology of Hearing (IFPS:/KB/7/2020) and conformed with the Declaration of Helsinki. All the patients’ parents/legal guardians signed an informed consent document upon entering the study.

### 2.2. Audiological Evaluation

All patients were assessed using audiological tests, including pure-tone audiometry with an Interacoustic AC40 (Interacoustic, Middelfart, Denmark) clinical diagnostic audiometer, unaided and aided hearing thresholds in a sound field (bone conduction hearing aid on a soft band), and a speech test in quiet and noise performed with an Aurical Aud1081 audiometer (Aurical, Taastrup, Denmark). The patients’ audiometric thresholds for air conduction, bone conduction, and in a sound field were assessed pre-operatively, during activation, and at 1 and 6 months post-operatively. Sound field thresholds were measured using warble tones presented from a loudspeaker situated 1 m in front of the subject. The word recognition score (WRS) was assessed with the Demenko and Pruszewicz Polish Monosyllabic Word Test at 65 dB SPL presented via a loudspeaker.

In a sound field the contralateral ear was double-blocked with an earplug. The non-implanted ear was blocked but not masked since masking would reduce the sensitivity of that ear to bone-conducted sound which is normally also heard from an implant on the other side of the skull. If there was an air-bone gap of more than 10 dB in the test ear (pure tone audiometry), masking was required. For bone-conduction, to mask the non-test ear we added 10 dB HL to the air conduction threshold in the non-test ear and added the occlusion effect (that is, air conduction in non-test ear +10 dB HL + occlusion effect). We used the plateau method in which the above formulas for BC and AC determined the starting masking level [28,29,30,31].

Speech reception thresholds in noise were assessed using the Polish Sentence Matrix Test (PSMT) with signal and noise presented from the front (S0–N0), with the noise level fixed at 65 dB SPL. The PSMT consists of five columns containing 10 names, 10 verbs, 10 numerals, 10 adjectives, and 10 nouns.

Prior to surgery, all patients were fitted with a reference device –a bone conduction hearing aid on a soft band—based on in-situ thresholds. The simulation was carried out to replicate the performance of the actual implant and evaluate the possible hearing benefits from implantation.

### 2.3. Survey Assessment

Patient’s satisfaction and quality of life were assessed before implantation and at 1 and 6 months post-operatively with the Children’s version of the Abbreviated Profile of hearing Aids Benefit (APHAB) (Abbreviated Profile of Hearing Aid Benefit) questionnaire. The Polish language version of the APHAB questionnaire was used. This questionnaire measures subjective hearing impairment on four different subscales pertaining to different listening situations and assesses what benefit the patient might expect from implantation [32,33].

### 2.4. Statistical Analysis

A Shapiro–Wilk test was used to check whether the analyzed variables were normally distributed. A repeated measures analysis of variance with Bonferroni adjustment for multiple comparisons was performed to test for changes in mean scores over several time points. The *p*-level was set as below 0.05. All statistical tests were performed using IBM SPSS Statistics24 (New York, NY, USA).

### 2.5. Surgery

A modified “C” retroauricular cut was made, and the subcutaneous tissues were displaced until the periosteum was reached. It was important to preserve the anterior auricular artery and the posterior auricular artery and vein. It sometimes happened that occipital vessels were in the line of surgery as well as the emissary vein; in such cases bone wax was used. Small blood vessels were coagulated. An opening was made in the mastoid in a place that was determined earlier from CT scans. When the periostium and subcutaneous tissue are laid out, this is the moment to put the template in place and assess whether the place in the bone is also good from a cosmetic point of view, since the BCI602 elevates the skin a little. Conditions should be suitable for glasses in the future and the skin should not be too tight. Ideally, the hole should not contact the dura or sigmoid sinus. If anatomical conditions are insufficient, there is the possibility of clearing out the bone over the dura or sigmoid sinus and preserving a thin plate of bone to secure the implant. After confirming that the implant fitted in the correct position, the operating cavity was rinsed and two holes were made through the template using a custom drill. The BCI602 was placed in the previously made cavity, and the plastic part of the implant bent through 0 or 90°. The implant was attached using two custom screws (there was no need to use lifts in either case). After checking that the implant was correctly positioned, subcutaneous and skin sutures were applied (Polysorb 3.0 and Monosoft 2.0) (Covidien, Dublin, Ireland). After the procedure, all patients were in good condition and received antibiotics intravenously twice daily as well as pain medication. After 3 days in hospital, all were discharged in good general condition, and received amoxicillin with clavulanic acid twice a day for 7 consecutive days with probiotic. The dressing as well as sutures were removed from the retroauricular incision 7–10 days after surgery [21,34].

## 3. Results

This study included 22 children with conductive or mixed hearing loss due to various medical reasons who underwent unilateral BCI 602 implantation. There were 9 girls and 13 boys aged 8 to18 years, and their mean age was 14.7 years (SD = 2.7). Of them, 15 had conductive and 7 had mixed hearing loss; 12 were implanted in the right and 10 in the left. The causes of hearing loss were as follows: microtia and atresia (*n* = 18); congenital defect of the middle and inner ear (*n* = 3); and chronic otitis media (*n* = 1).

### 3.1. Pure Tone Audiometry

**Figure 4:**
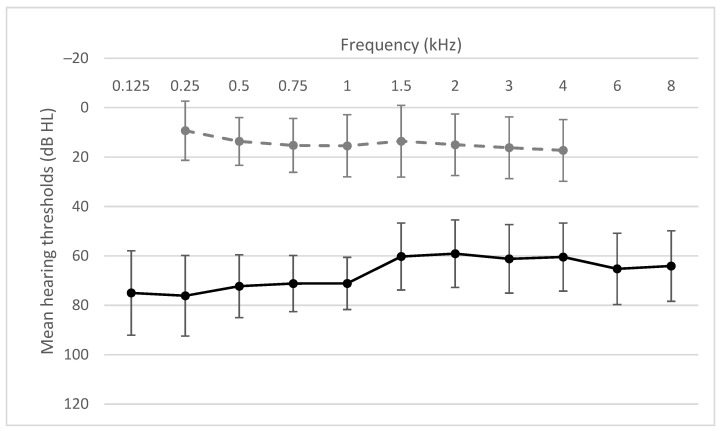
Air conduction thresholds (black solid line) and bone conduction thresholds (gray dashed line).

During the qualification process, we selected only healthy ears (without active infection). Before implantation, pure tone audiometry for air conduction was performed at octave frequencies (0.25–8 kHz) and results ranged from 46.3 to 88.8 dB HL (M = 65.7, SD = 9.9). Pure tone audiometry for bone conduction (0.25–4 kHz) ranged from 2.5 to 35 dB HL (M = 15.3, SD = 10.5). Detailed data are shown in Figure 4.

### 3.2. Functional Gain

**Figure 5:**
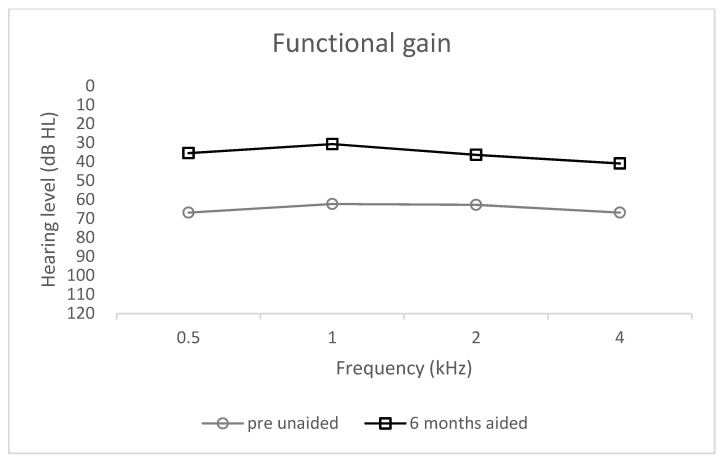
Functional gain; unaided and 6 months aided.

The mean functional gain estimated in the simulation before implantation was 31.8 dB HL (SD = 10.5). The real mean functional gain after 6 months compared to before implantation was 28.8 dB HL (SD = 28.8). The functional gain were comparable at each of the individual frequencies (Figure 5).

### 3.3. Sound Field Thresholds

**Figure 6:**
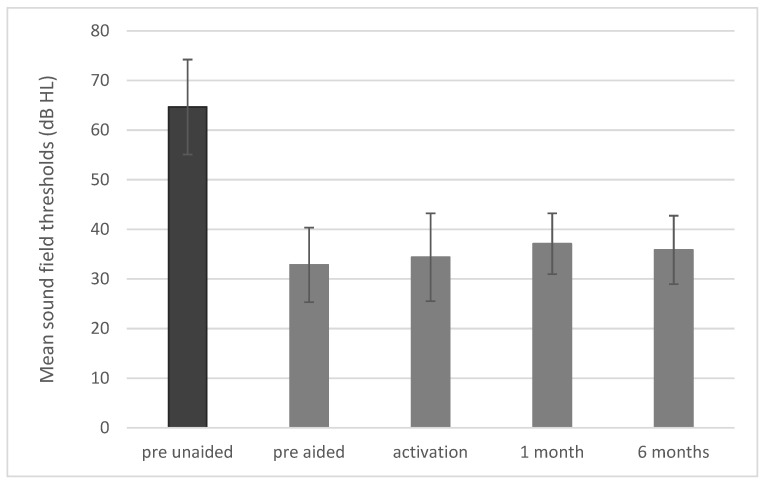
Sound field thresholds. All differences between pre-unaided and all other conditions are statistically significant at *p* < 0.001. The bars represent mean scores, the error bars are standard deviations.

The mean hearing threshold in a sound field before implantation was 64.7 dB HL (SD = 9.6) and in the simulation it was 32.8 dB HL (SD = 7.5). A statistically significant improvement was observed after surgery (F = 82.78; *p* < 0.001; e^2^ = 0.80). The mean hearing threshold was34.4 dB HL (SD = 8.9) upon activation, 37.1 dB HL (SD = 7.5) after 1 month, and 35.9 dB HL (SD = 7.5) after 6 months (Figure 6).

### 3.4. Free-Field Word Recognition in Quiet

**Figure 7:**
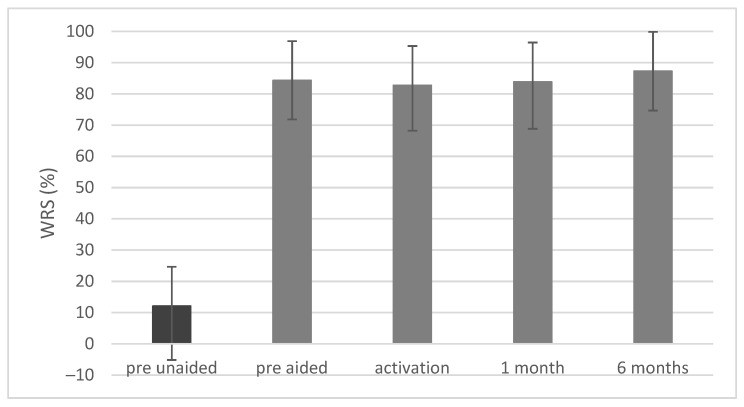
Word recognition scores (WRSs) in quiet obtained in free-field audiometry at 65 dB SPL. All differences between pre-unaided and all other conditions are statistically significant at *p* < 0.001. The bars represent mean scores, the error bars are standard deviations.

Before implantation the mean word recognition score (WRS) measured via headphones was 7.3% (SD = 17.6). Unaided, the scores were 12.1% (SD = 17.2) and aided (in the simulation) 84.3% (SD = 12.5). After surgery a statistically significant improvement was observed (F = 122.19; *p* < 0.001; e^2^ = 0.85). Upon activation the mean WRS was 82.7% (SD = 14.5); 1 month later it was83.9% (SD = 15.0) and after 6 months it was 87.3% (SD = 12.6) (Figure 7).

### 3.5. Intelligibility of Speech in Noise

**Figure 8:**
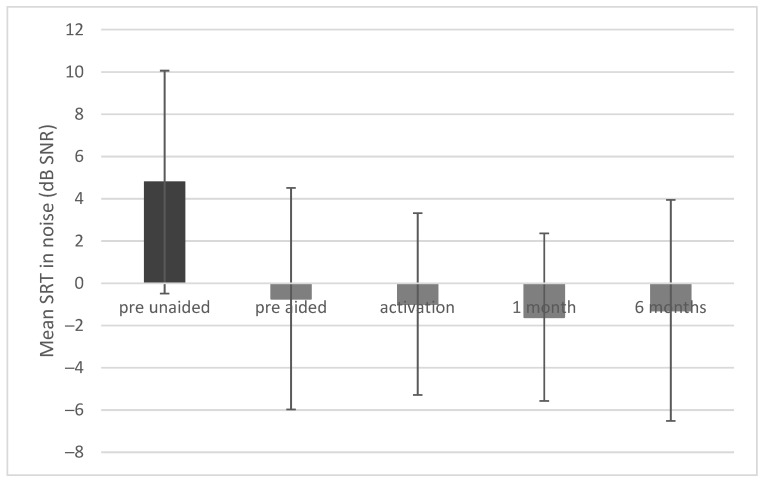
Speech reception thresholds (SRTs) in noise. All differences between pre-unaided and all other conditions are statistically significant at *p* < 0.001. The bars represent mean scores, the error bars are standard deviations.

Before implantation the mean speech reception threshold (SRT) was 4.79 dB SNR (SD = 5.28) unaided and −0.73 dB SNR (SD = 5.24) aided (in the simulation). After surgery a statistically significant improvement was observed (F = 10.90; *p* < 0.001; e^2^ = 0.35). The mean SRT was −0.98 dB SNR (SD = 4.30) upon activation, −1.61 dB SNR (SD = 3.97) after 1 month, and −1.29 dB SNR (SD = 5.23) after 6 months (Figure 8).

### 3.6. Patient-Reported Outcomes

**Figure 9:**
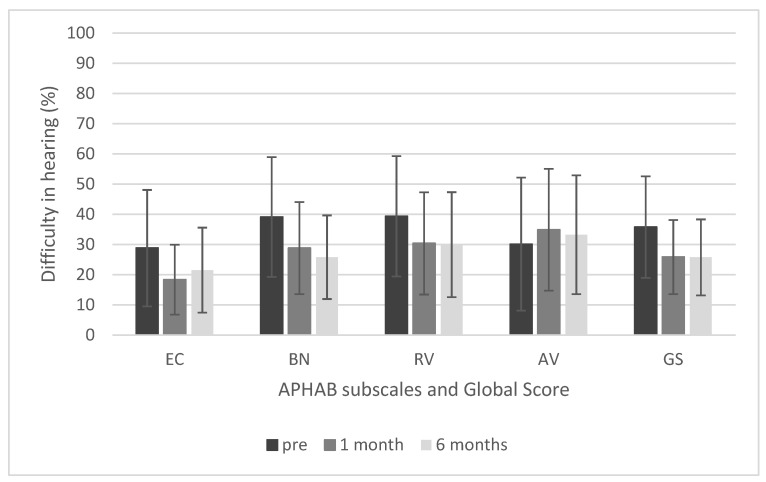
Results of APHAB questionnaire.EC, Ease of Communication; BN, Background Noise; RV, Reverberation; AV, Aversiveness; GS, global score. The differences in the EC, BN, and RV subscales are statistically significant at *p* < 0.05, but the difference in the AV subscale is not. The bars represent mean scores, the error bars represent standard deviations.

The APHAB questionnaire showed that difficulties in hearing decreased after implantation. There was a statistically significant improvement in the global score (F = 9.35; *p* < 0.001; e^2^ = 0.32), and the pre-operative score (M = 35.7; SD = 16.8) was significantly higher than the post-operative score at1 month (M = 25.8; SD = 12.3) and 6 months (M = 25.7; SD = 12.6). The same was true for the Reverberation (RV) subscale (F = 7.56; *p* < 0.002; e^2^ = 0.27). In the Ease of Communication (EC) subscale, a significant change (F = 4.46; *p* = 0.018; e^2^ = 0.18) was observed between the pre-operative score and the 1-month follow-up. For the Background Noise (BN) subscale, a significant change (F = 7.52; *p* < 0.002; e^2^ = 0.27) was found between the pre-operative score and the 6-month follow-up. On the Aversiveness (AV) subscale the results across all three time points remained stable (F = 0.64; *p* = 0.532). Mean APHAB scores are shown in Figure 9.

## 4. Discussion

In the case of pediatric patients, the most important aspect when choosing an implantable device to improve hearing is its effectiveness and, above all, a safe surgical procedure. Our study is consistent with the existing literature, underlining the effectiveness of an active bone conduction device, especially in challenging conditions such as in children with a congenital defect of the outer and middle ear. The Bonebridge implant surgery is safe for pediatric patients who experience various anatomical limitations [35,36,37,38]. The transcutaneous BC implants, including the BCI602, avoid the drawbacks associated with percutaneous implants such as skin reactions, growth of skin over the abutment, implant extrusions, wound infections, and fixture losses. This is why the complication rate is higher with percutaneous BC implants in comparison with transcutaneous ones.

Moreover, the Bonebridge has a much lower complication rate than the BAHA implant (24%), which is associated with a higher rate of revision surgery (12%). In long-term follow-up of BAHA use (mean 14 years), reported figures have been a skin reaction rate of 31%, loss of osseointegration in 17%, and need for revision surgery in 34% [13,15,16,17].

In this study the 6-month follow-up showed no postoperative problems. Postoperative wounds healed properly, and no pain or infections appeared.

From a surgical point of view, no case was canceled due to mastoid size. For cases where there is no atresia or microtia, a superior–posterior C-shaped cut is recommended. because the device can increase the skin contour and elevate the skin. If a typical S-shaped cut is performed, there is a chance that the skin at the anterior edge of the device could become so thin that there is the potential risk of extrusion. In cases after pinna reconstruction, the cut should be performed in line with the scar (according to vascular conditions). An additional factor is proper location on the mastoid, which should be considered if there is a choice between the BB602 and BB601. If the surface of the mastoid is rather curved and there is the opportunity to better adjust the implant, the authors recommend locating the device in a more superior position. It can even be placed more temporally. If there is thickening of the bone, there can sometimes be the need to adjust placement of the implant so that the dura is pressed a little against the thin bony plate. There is also the possibility of placing wax on the bottom of the cavity. Wax can also be used in cases where there is contact with the middle ear cavity through an aerated mastoid; wax prevents aeration of subcutaneous tissue from the tympanic cavity. Incases where there is risk of improper healing due to poor quality of the surface of the temporal bone, there is possibility to keep through 1-day redon drainage with vacuum.

This analysis of 22 cases of implantation of the BonebridgeBCI602 in children in our department confirms that it provides significant audiological benefit. From an audiological point of view, the most important factor confirming the effectiveness of the device is the improvement in speech understanding. This is especially important for children experiencing difficult acoustic conditions. The mean word recognition score (WRS) changed from 12.1% before implantation to 84.3% at activation, then to 83.9% 1 month after activation and finally to 87.3% after 6 months. All patients showed stable postoperative results. The improvement in speech understanding seen in our research is comparable to the results of previous studies [21,34,39,40]. For example, Bravo-Torres et al. reported an improvement in speech recognition from 29.4% before surgery to 96.4% after wards, and Zernotti et al. reported an improvement in speech understanding from 29.4 to 97% 1 month after activation.

Our study also assessed speech understanding in noise, which is especially important for children. Mean speech reception threshold (SRT) before implantation was +4.79 dB SNR and improved to −1.29 dB SNR after 6 months. Some studies have also measured hearing in noise [34,40,41,42]; as in our work, all authors have reported a benefit with the use of the Bonebridge and have seen better speech understanding in noise. The audiological gain of the Bonebridge has been reported by many workers [34,40,41,42,43,44]; in this study the real mean functional gain after 6 months compared to before implantation was 28.8 dB HL. The mean hearing threshold in a sound field before implantation was 64.7 and 35.9 dB HL after 6 months.

Studies often use survey tools to assess the subjective benefits in daily functioning after implantation of a Bonebridge device. As found in this current work, all studies confirm that the auditory benefits positively affect the patient’s quality of life [6,21,34,44]. The APHAB questionnaire applied in our work showed that difficulties in hearing decreased after implantation, with a statistically significant improvement in global score. Pre-operative scores (M = 35.7) were significantly worse than post-operative scores at 1 month (M = 25.8) and 6months (M = 25.7). Interestingly, despite some poor audiological results, the children did not report major problems in the questionnaires. This may be due to the current pandemic situation: COVID-19 has led to more remote learning, limited contacts with peers, and fewer difficult acoustic situations. From a surgical point of view, the placement of the floating mass transducer of the BCI602 requires less bone removal. The findings of Wenzel et al. confirm that changes in the dimensions of the internal part of the BCI602 benefit patients in all age groups and with all indications. According to prior authors, placement of the BCI602 requires less bone removal than the BCI601, and “the newer BCI 602 transducer is more likely than its predecessor to be completely accommodated in the mastoid bone” [45]. In our experience, the flexible design and the transition between the receiver coil and the floating mass transducer makes it possible to properly position the implant even in difficult anatomical conditions. In addition, the reduced drilling depth allows a wider group of patients, who previously were unable to use the first generation device due to limited anatomical conditions, to be accommodated. Finally, the self-tapping screws and smaller size reduce the risk of damage to the meninges and reduce surgery time.

## 5. Conclusions

Our study showed a significant improvement in hearing after treatment with BonebridgeBCI602 implant. Although the results were below normal in the free field, the patients obtained very good results of speech understanding after implantation. Without the implant, speech understanding was very challenging especially in more difficult acoustic conditions.

The surgery is safe and the device is effective among children with congenital ear defects suffering from conductive or mixed hearing loss. The audiological results show a significant improvement in hearing and speech understanding in quiet and noise. Subjectively, patients experience the benefits—notably an improvement of hearing—in everyday conditions. The second-generation of Bonebridge provides an opportunity for patients with limited or difficult anatomical conditions who previously found it impossible to use the first-generation device. Preoperative planning with a CT is still recommended for all children.

Improving hearing could be more sufficient for such population. We believe that in future even more powerful devices will be accessible with possibility of improvement for patients with moderate hearing loss. Especially with mixed hearing loss and progressive loss it is a challenge. There is always risk for some decrease of hearing bone conductive thresholds so each surgery should be considered meticulously.

## Data Availability

All relevant data are within the manuscript. Data supporting publication according to institutional regulation is available on demand for potential stakeholder.
